# Infection of guppies (*Poecilia reticulata*) with the Asian fish tapeworm *Schyzocotyle acheilognathi* in an urban stream in Brazil

**DOI:** 10.1590/S1984-29612024018

**Published:** 2024-04-05

**Authors:** Jordana Costa Alves de Assis, Hudson Alves Pinto

**Affiliations:** 1 Laboratório de Biologia de Trematoda, Departamento de Parasitologia, Instituto de Ciências Biológicas, Universidade Federal de Minas Gerais – UFMG, Belo Horizonte, MG, Brasil

**Keywords:** Brazil, cestodes, fish, helminths, Poecilia reticulata, Schyzocotyle acheilognathi, Brasil, cestoides, peixe, helmintos, Poecilia reticulata, Schyzocotyle acheilognathi

## Abstract

*Schyzocotyle acheilognathi* is a fish tapeworm native to Asia but has been reported as an alien species on practically all other continents. Its invasive potential is due to its low host specificity and high adaptability to different environments, and its spread to new areas can result in economic and ecological impacts. Studies reporting this species in South America are still scarce, indicating the need to monitor its dispersion to new areas. Herein, tapeworms found in guppies, *Poecilia reticulata*, from an urban stream located in Belo Horizonte, Minas Gerais, Brazil, in April 2021 were subjected to morphological and molecular characterization. As a result, 5/13 (38.5%) of the *P. reticulata* specimens evaluated were infected with intestinal tapeworms. It was verified a mean intensity of infection of 7.8 (1-25) and a mean abundance of infection of 3 (0-25). The morphology of the cestodes obtained was compatible with that of *S. acheilognathi*. Moreover, genetic analysis based on cytochrome c oxidase subunit 1 gene (*Cox-1*) revealed 97.89-99.77% similarity to isolates of this species from different localities. The possibility that *S. acheilognathi* is expanding to new regions of South America is discussed.

## Resumo

*Schyzocotyle acheilognathi* é um cestoide de peixes nativos da Ásia, mas tem sido reportado como uma espécie exótica em praticamente todos os demais continentes. Seu potencial invasor ocorre devido à sua baixa especificidade por hospedeiros, assim como alta adaptabilidade a distintos ambientes. Sua dispersão para novas áreas pode resultar em impactos econômicos e ecológicos. Estudos que relatam essa espécie na América do Sul ainda são escassos, o que indica a necessidade de monitorar sua dispersão para novas áreas. Neste trabalho, cestoides encontrados em lebistes, *Poecilia reticulata*, de um córrego urbano, localizado em Belo Horizonte, Minas Gerais, Brasil, em abril de 2021, foram submetidos à caracterização morfológica e molecular. Como resultado, 5/13 (38,5%) dos espécimes de *P. reticulata* avaliados estavam infectados com cestoides intestinais. Verificou-se uma intensidade média de infecção de 7,8 (1-25) e uma abundância média de infecção de 3 (0-25). A morfologia dos cestoides obtidos foi compatível com a de *S. acheilognathi*. Além disso, a análise genética, baseada no gene citocromo c oxidase subunidade 1 (*Cox-1*), revelou 97,89-99,77% de similaridade com isolados dessa espécie provenientes de diferentes localidades. Dessa forma, discute-se a possibilidade de *S. acheilognathi* estar se expandindo para novas regiões da América do Sul.

The Asian fish tapeworm (AFT), *Schyzocotyle acheilognathi* (Yamaguti, 1934), is native to East Asia; it is currently considered one of the most successful invasive parasites and has been reported on practically all continents except Antarctica (Scholz, 1997; Choudhury et al., 2006; Pérez-Ponce de Léon et al., 2018; Kuchta et al., 2018). The successful introduction and dispersion of this parasite are facilitated by its low specificity for definitive and intermediate hosts, by its adaptability to different climates (ranging from tropical to colder), and by human action, especially the intense commercial translocation of infected fish (Choudhury et al., 2006; Pérez-Ponce de León et al., 2018; Kuchta et al., 2018; Boonthai et al., 2017). The introduction of AFT into new areas is a concern, given that these cestodes have been associated with direct injuries to the fish gastrointestinal tract, such as intestinal wall distension, inflammation, degeneration, and cell necrosis (Britton et al., 2011; Scholz et al., 2021). Moreover, indirect changes in the intestinal microbiota (Boonthai et al., 2017) can be deleterious to infected fish. All these findings serve as warnings about the negative pathogenic impacts of *S. acheilognathi* on fish health worldwide. To date, only three studies in South America have reported AFT in fish in Brazil (Rego et al., 1999; Santos et al., 2017; Souza et al., 2018), and one has reported AFT in Argentina (Waicheim et al., 2014). However, the possibility that this alien parasite is more widely distributed on this continent cannot be ruled out. Therefore, this study aims to report the occurrence of *S. acheilognathi* in a new region of South America, as well the possible factors involved in its dispersal.

**Figure 1:**
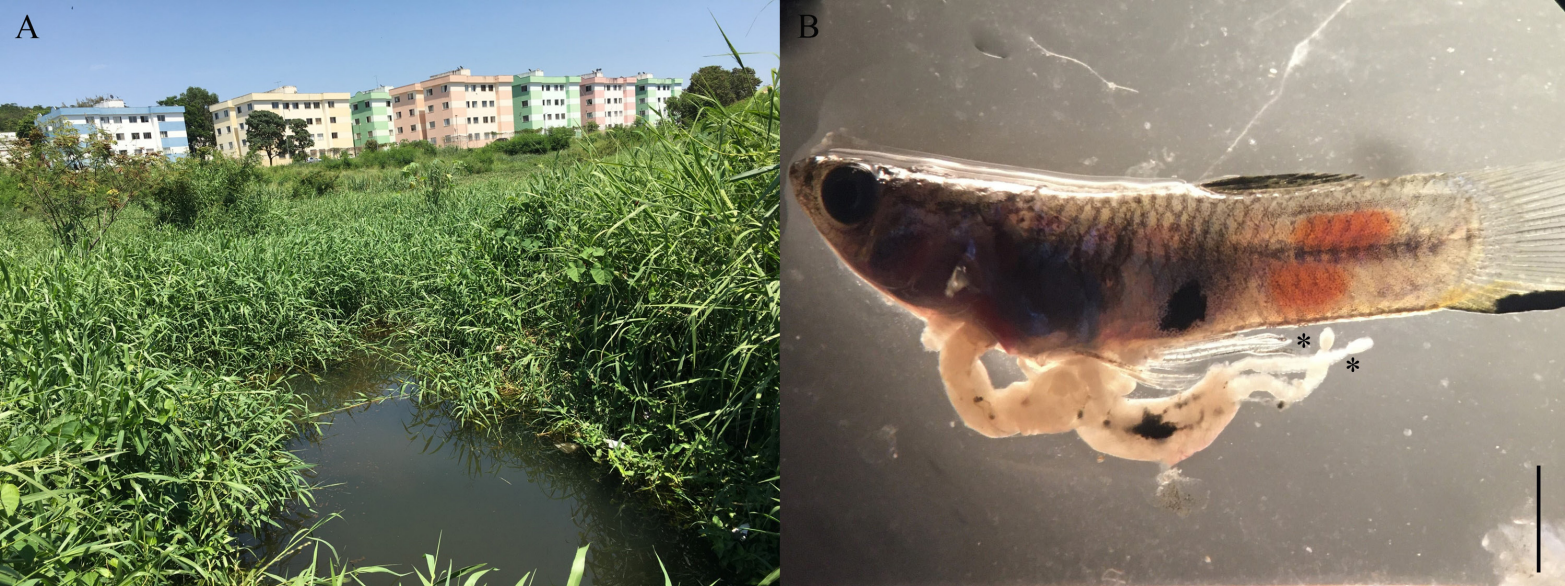
The finding of *Poecilia reticulata* infected with tapeworms in Brazil. A) Photo of the stream located in an urban area in Belo Horizonte, Minas Gerais State, where fish were found to be infected in April 2021; B) A fish specimen with two worms (indicated by asterisks) emerging from the intestine sectioned during necropsy. Scale bar: 3 mm.

In the present study, during a malacological survey carried out in the city of Belo Horizonte, Minas Gerais, Brazil, in April 2021, 13 adult specimens of *Poecilia reticulata* (Peters, 1860) (Poeciliidae) measuring 1-3 cm in total length (length mean 2 cm) were accidentally collected and mixed with organic matter and other small aquatic organisms. The sampling site (19°52'04.0" S 43°57'09.8" W) is in an urbanized area of the city next to a housing complex (Figure 1A). This waterbody is characterized by a stream that runs through a neighborhood and an ecological park where a rich diversity of animals, such as mollusks, fish and birds, can be observed. In the laboratory, the fish were found dead, separated from the organic material collected and washed in tap water. Later, the specimens were dissected under a stereomicroscope to search for helminths, which revealed the infection with tapeworms (Figure 1B). These helminths were collected, counted, ringed in 0.7% saline solution, and fixed in hot 10% formalin. For the morphological study, the worms were stained with alum aceto-carmine and processed through an ethanol dehydration series, followed by diaphanization with beechwood creosote. Later, the worms were mounted on permanent slides in Canada balsam. The slides were examined using the Leica DM500 and DM750 microscopes with differential interference contrast (DIC). Photographs were taken with the aid of a Leica ICC50 HD digital camera. The morphology and measurements of the specimens evaluated were compared with descriptions available in works by different authors (Yamaguti, 1934; Scholz, 1997; Rego et al., 1999). The ecological terms used were established in Bush et al. (1997).

For molecular analysis, a small fragment was sectioned at the posterior extremity and fixed in 95% ethanol. DNA extraction was performed with the QIAamp^®^ DNA Mini Kit. A partial region of the *Cox-1* gene was amplified by PCR using the primers JB3 and JB4.5 (Bowles et al., 1992). The PCR reagents and conditions were previously described by our research group (Assis et al., 2019). Amplicons were sequenced in both directions in an ABI 3730 capillary sequencer using a POP7 polymer and Big Dye v3.1 Terminator Cycle Sequencing Ready Reaction Kit. The contig was generated in ChromasPro version 2.0.1 and subjected to a BLAST search. An alignment was constructed in MEGA 7.0 and used for the construction of a pairwise genetic distance matrix. The molecular sequence generated in this study was deposited in the GenBank database under the accession number PP210023.

**Figure 2:**
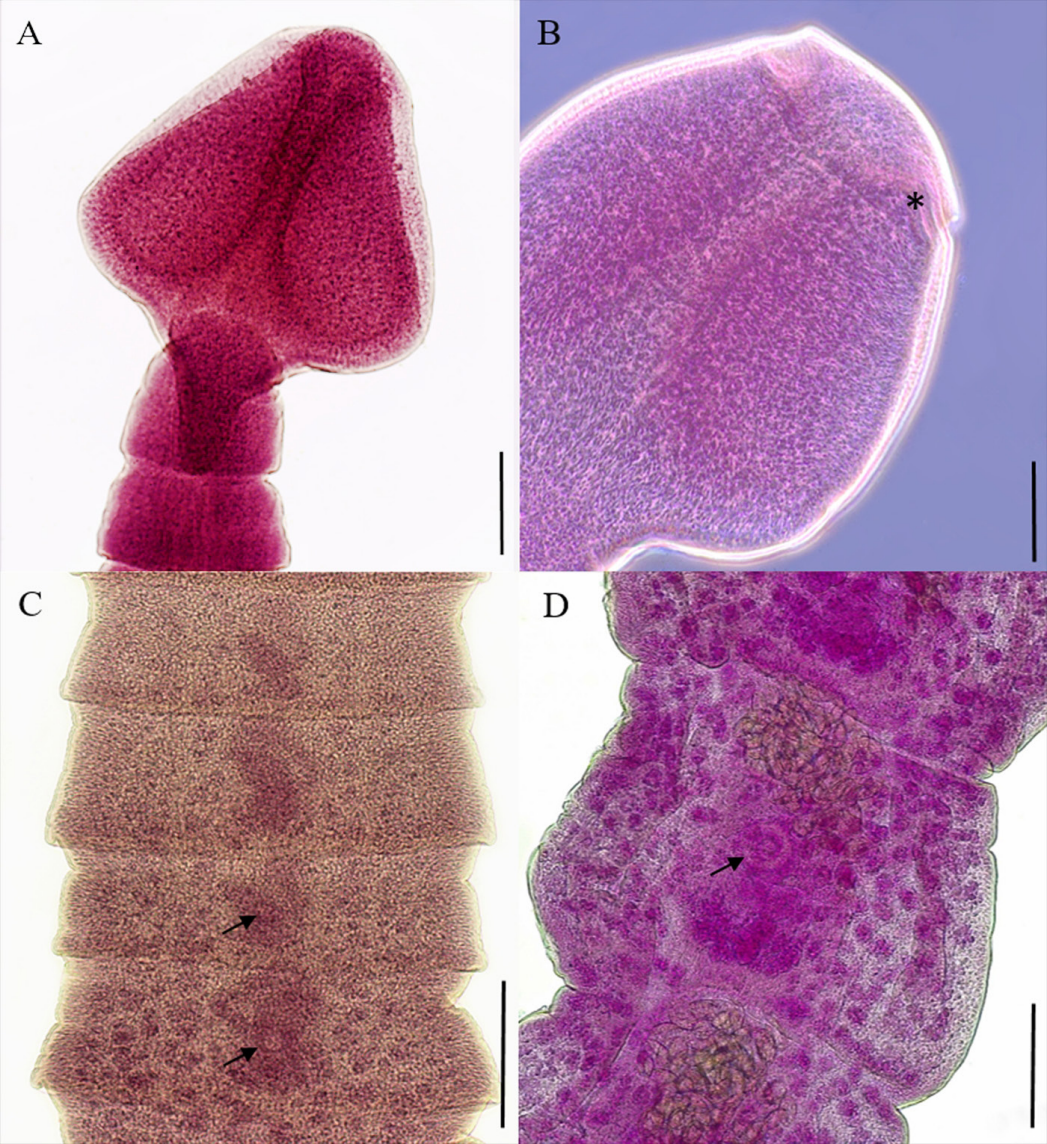
*Schyzocotyle acheilognathi* was found in free-living *Poecilia reticulata* from Brazil. A) Scolex with two bothria; B) Apical disc (asterisk); C) Strobilus - mature proglottid; D) Strobilus – gravid proglottid (genital pore indicated by arrows). Scale bars: A, C and D-200 µm, B-100 µm.

Five out of the 13 (prevalence of infection: 38.5%) specimens of *P. reticulata* examined were found to be infected with intestinal tapeworms (fish measuring 1.5-2 cm in total length, 1.8 cm length mean). In total, 42 worms were recovered. The mean abundance of infection and the mean intensity of infection were 3 (0-25) and 7.8 (1-25), respectively. Ten worms from different hosts were subjected to morphological analysis. The strobilus is elongated, 9.3 (5-14) mm. The scolex, 637 (530-716) μm in length and 672 (580-754) μm in width, is pyramidal to rounded-shaped, with a thin apical disc, two bothria with discrete openings, (Figure 2A-B). The mature proglottids had numerous medullary rounded testes, 50-76 in number and 32 (23-41) μm in length and 36 (28-48) μm in width, and cortical and confluent rounded vitelline follicles with 29 (19-48) μm in length and 25 (18-30) μm in width. Ovary lobated, at the posterior margin of proglottids, with 116 (76-171) μm in length and 216 (174-261) μm in width. The genital pore is median (Figure 2C). Gravid proglottids were present (Figure 2D). The eggs were operculated, unembryonated, yellow in color, and 55 (45-64) μm in length and 31 (25-38) μm in width. Overall, the morphology of the cestodes characterized here was in agreement with that described by different authors for *S. acheilognathi* (Yamaguti, 1934; Scholz, 1997; Rego et al., 1999). 

In the genetic analysis, a 426 bp fragment of the *Cox-1* gene was obtained. A comparison with data available in GenBank revealed 97.89-99.77% similarity to five *S. acheilognathi* isolates found in fish from different countries [99.77% similarity to worms found in *Ctenopharyngodon idella* from China (KX589243); 98.59% similarity to worms found in *Paretroplus kieneri* from the United Kingdom (MG968746); 98.59% similarity to worms found in *Cyprinella lutrensi* from the USA (KX060595); 98.59% similarity to worms found in *Cyprinus carpio* from Turkey (MN369445); and 97.89% similarity to worms found in *Labeobarbus nedgi* from Ethiopia (KX060588)]. Regarding the other representative species of the genus *Schyzocotyle* with available genetic data for comparison, i.e., *Schyzocotyle nayarensis* (Malhotra, 1983), the similarity to our Brazilian isolate of *S. acheilognathi* was relatively low [86.85% similarity to the two Indian isolates found in *Barilius* sp., KR780829 and *Raiamas bola*, NC030317] (Supplementary material - Table S1). Currently, the mitochondrial marker *Cox-1* has been widely used in phylogenetic and epidemiological studies of cestodes of the order Bothriocephalidea, such as *S. acheilognathi*, composing a database with isolates from different locations on the world (Kuchta et al., 2018; Pérez-Ponce de León et al., 2018). Although this AFT has been reported in South America, the present study contributes with unprecedented genetic data for the continent.

This work represents the first report of *P. reticulata* infected with *S. acheilognathi* in Brazil. Moreover, the first molecular data for a South American isolate of AFT are presented. The new record of AFT in free-living fish provides some warnings about the possibility of spreading this invasive parasite on this continent. In Brazil, thus far, there are three records of *S. acheilognathi*, all of which are in the Southern Region of the country. The first report was based on worms obtained from cultivated carp (*C. carpio*) from a fish farm in Paraná State (Rego et al., 1999). Several years later, Santos et al. (2017) reported the same fish species infected with AFT in Santa Catarina State. More recently, *S. acheilognathi* was found in a free-living native species, *Rineloricaria pentamaculata,* by Souza et al. (2018) in the state of Santa Catarina. According to these reports, the prevalence of infection was lower than that reported here (30/229, 13%; 1/15, 6.6%), but research focusing on the health of fish in the presented stream here is necessary. In the present study, the occurrence of this invasive cestode in the Southeastern Region of Brazil was reported for the first time, which suggested that this disease can spread throughout the country. In this regard, although there are few reports of infection by *S. acheilognathi* in South America, there are no monitoring protocols being implemented in this region, which leads us to infer that there may be underreporting of this parasite on the continent. Notably, AFT has already been a problem for cultivated and free-living fish fauna in some localities of Central America and North America (Salgado-Maldonado & Pineda-López, 2003; Choudhury et al., 2006; Kuchta et al., 2018; Pérez-Ponce de Léon et al., 2018; Scholz et al., 2021). Thus, this problem has been neglected in the context of aquaculture in South America.

Interestingly, the aquatic collection site discussed here is located in a green area close to a residential complex with no history of fish farming practices in a metropolis with more than 2 million inhabitants. This fact raises questions regarding the establishment of AFT at this locality. Its introduction through aquarium practices may be a possible explanation since infections caused by this tapeworm in ornamental fish farming have already been reported in Brazil (Santos et al., 2017). *Schyzocotyle acheilognathi* was previously found in an ornamental *C. carpio* purchased at an aquarium store from Belo Horizonte approximately ten years ago (HAP, personal communication). In this respect, the introduction of *S. acheilognathi* in new countries has been directly associated with human action, especially the translocation of infected fish without effective sanitary control (Salgado-Maldonado & Pineda-López, 2003; Choudhury et al., 2006; Pérez-Ponce de Léon et al., 2018; Kuchta et al., 2018). Thus, finding the parasite in free-living fish from an urban waterbody is worrying since dispersal to new areas may be favored in a way unrelated to aquarism.

*Poecilia reticulata* is native to northwestern South America and is native to the Guayre River in Caracas, Venezuela (Rosen & Bailey, 1963). This fish is omnivorous and lives in several systems, ranging from fresh to brackish water, including those with anthropic alterations (Araújo et al., 2009; Torres-Dowdall et al., 2013). Over time, the species has been used in ornamental fish farming, as well as for potential biological control of mosquito larvae (Seng et al., 2008; Shafique et al., 2019). Its high reproduction rate and resistance to entropized environments favor its indiscriminate dispersal (Seng et al., 2008; Shafique et al., 2019; Dias et al., 2020). Although several helminth parasites have been reported in *P. reticulata* in Brazil (Kohn et al., 2007; Tavares-Dias et al., 2009; Pinto & Melo, 2011), infection with AFT is reported here for the first time. This poecilid species was previously reported to be infected with *S. acheilognathi* in Guatemala and México (Scholz, 1997; Pérez-Ponce de Léon et al., 2018). In the latter, 15 poecilid species have been reported to be infected in different locations over time (revised by Pérez-Ponce de Léon et al., 2018). Therefore, the present study provides insight into the expansion of this parasitosis to other regions of South America. Thus, monitoring strategies to prevent potential impacts caused by AFT on pisciculture and especially on native fish fauna are necessary, as research development about diagnosis and treatment of AFT infection in commercial fish farming and translocation control of fish from regions with records of cestode to free regions. 

## Supplementary Material

Supplementary material accompanies this paper.

Table S1 Pairwise comparison between *Cox-1* sequences (426bp) *Schyzocotyle acheilognathi* from obtained from Brazil and data available to isolates of *Schyzocotyle* spp.

This material is available as part of the online article from https://doi.org/10.1590/S1984-29612024018
