# Supplementary material for: Infection of guppies (Poecilia reticulata) with the Asian fish tapeworm Schyzocotyle acheilognathi in an urban stream in Brazil
Source: Rev Bras Parasitol Vet. 2024 Apr 5;33(1):e020323. doi: 10.1590/S1984-29612024018 (PMC11020102; doi:10.1590/S1984-29612024018)
Supplement: Table S1 [file rbpv-33-1-e020323-Suppl.pdf]

Table S1. Pairwise comparison between *Cox-1* sequences (426bp) *Schyzocotyle acheilognathi* from obtained from Brazil and data available to isolates of *Schyzocotyle* spp.

	**Species**	**GenBank Accession Number**	**Hosts**	**Locality**	**Molecular Similarity (%)**						
(1)	*S. acheilognathi*	Present study	*Poecilia reticulata*	Brazil							
(2)	*S. acheilognathi*	KX589243	*Ctenopharyngodon idella*	China	99.77						
(3)	*S. acheilognathi*	MG968746	*Paretroplus kieneri*	United Kingdom	98.59	98.83					
(4)	*S. acheilognathi*	KX060595	*Cyprinella lutrensi*	USA	98.59	98.83	100.00				
(5)	*S. acheilognathi*	MN369445	*Cyprinus carpio*	Turkey	98.59	98.83	100.00	100.00			
(6)	*S. acheilognathi*	KX060588	*Labeobarbus nedgi*	Ethiopia	97.89	98.12	98.36	98.36	98.36		
(7)	*S. nayarensis*	NC030317	*Raiamas bola*	India	86.85	87.09	87.09	87.09	87.09	86.62	
(8)	*S. nayarensis*	KR780829	*Barilius* sp.	India	86.85	87.09	87.09	87.09	87.09	86.62	100.00

Reference

(1) Present study.

(2) Li WX, Zhang D, Boyce K, Bing WX, Hong Z, Wu SG, et al. The complete mitochondrial DNA of three monozoic tapeworms in the Caryophyllidea: a mitogenomic perspective on the phylogeny of eucestodes. *Parasit Vectors* 2017; 10.1186/s13071-017-2245-y.

(3) Kuchta R, Choudhury A, Scholz T. Asian Fish Tapeworm: The most successful invasive parasite in freshwaters. *Trends Parasitol* 2018; 34(6): 511-523.

(4, 8) Brabec J, Waechenbach A, Scholz T, Littlewood D, Timothy J, Kuchta R. Molecular phylogeny of the Bothriocephalidea (Cestoda): molecular data challenge morphological classification. *Int J Parasitol* 2015; 45: 761-771.

(5) Not published.

(6, 7) Brabec, J., Kuchta, R., Scholz, T., Littlewood, D.T., 2016. Paralogues of nuclear ribosomal genes conceal phylogenetic signal within the invasive Asian fish tapeworm lineage: evidence from next generation sequencing data. Int. J. Parasitol. 46: 555-562.
